# Phenolic Rich Fractions from Mycelium and Fruiting Body of *Ganoderma lucidum* Inhibit Bacterial Pathogens Mediated by Generation of Reactive Oxygen Species and Protein Leakage and Modulate Hypoxic Stress in HEK 293 Cell Line

**DOI:** 10.1155/2018/6285615

**Published:** 2018-12-17

**Authors:** Jigni Mishra, Anivesh Joshi, Rakhee Rajput, Kaushlesh Singh, Anju Bansal, Kshipra Misra

**Affiliations:** Defense Institute of Physiology and Allied Sciences, Delhi 110054, India

## Abstract

*Ganoderma lucidum* (*G. lucidum*) fungus (Family Ganodermataceae) is widely used as a traditional medicine in China, Japan, and many Asian countries on account of its numerous medicinal properties such as antioxidant, anticancer, antimicrobial, energy enhancing, and immunostimulatory. This broad spectrum of therapeutic effects exhibited by *G. lucidum* is ascribed to its abundance in several classes of chemical constituents, namely, carbohydrates, flavonoids, minerals, phenolics, proteins, and steroids which possess substantial bioactivities. The aim of the current study was to prepare phenolic rich fractions (PRFs) from aqueous extract of the Indian variety of *G. lucidum* mycelium and fruiting body. These fractions were assessed for their antioxidant capacity by TPC (total phenolic content), TFC (total flavonoid content), FRAP (ferric reducing antioxidant power), and ABTS [2,2-azino-bis(3-ethylbenzothiazoline)-6-sulfonic acid] assays. Quantification of flavonoids and nucleobases present in the fractions was carried out by high-performance thin layer chromatography (HPTLC). The antibacterial activity of the fractions was evaluated against *Escherichia coli*, *Salmonella typhi*, and *Staphylococcus aureus*. The antibacterial mechanism of action of the PRFs was established to be generation of reactive oxygen species and leakage of proteins within bacterial cells. Additionally, the protective effect of the PRFs in counteracting hypoxia was observed in HEK 293 cell lines.

## 1. Introduction

Modern medicine is witnessing a paradigm shift, with the focus being majorly centered on herbal sources as an alternative to synthetic medicines. Herbs comprise innumerous active metabolites which are capable of a vast array of pharmacological and therapeutic effects. Such metabolites include alkaloids, flavonoids, phenolics, terpenoids, carbohydrates, and esters. [1]. Among these bioactive constituents, phenolic compounds extracted from medicinal herbs and dietary plants have been reported to possess antioxidant, anticarcinogenic, anti-inflammatory, etc. effects [2]. Natural sources of medicinal importance encompass Kingdom Fungi which comprises multiple species of highly diverse and ubiquitous organisms that abundantly contain bioactive primary and secondary metabolites. These bioactive metabolites, either alone or in conjugation with other compounds, have been proven to promote human health owing to their multifarious pharmacological effects [3]. One such important medicinal mushroom among fungi is *Ganoderma lucidum* (*G. lucidum*) which has been documented in traditional Chinese medicine and traditional Tibetan medicine to be a highly effective therapeutic. Ancient oriental medical literature has reported the use of *G. lucidum* for its significant revigorating and energy-enhancing properties [4].

*G. lucidum* is a wood-rot basidiomycete classified within the family Ganodermataceae of Polyporales order. Its constituent bioactive compounds and their associated therapeutic properties against a number of health disorders have globally established *G. lucidum* as a pharmacologically and commercially important medicinal mushroom [5]. For instance, polysaccharides like ganoderans A, B, and C were reported to exhibit anticancer, anti-inflammatory, antimicrobial, and immunomodulatory effects [6]. Triterpenes present in *G. lucidum* were found to enhance its medicinal properties like antioxidant and lipid-lowering effects [7, 8]. HPTLC analysis performed on aqueous, ethyl acetate, and methanolic extracts of *G. lucidum* fruiting body and mycelia proved the presence of antioxidant compounds viz. ascorbic acid, gallic acid, rutin, and quercetin [9]. A study performed on different solvent (acetone, chloroform, methanol, and aqueous) extracts of *G. lucidum* mycelium showed encouraging results against pathogenic bacterial growth [10], which was mainly attributed to its phenolic contents. Similar study done on hexane, dichloromethane, ethyl acetate, and methanolic extracts of *G. lucidum* shed light on its antioxidant and antimicrobial efficacy [11].

In light of the aforementioned therapeutic potential of *G. lucidum*, the present study was undertaken as a novel attempt to characterize phenolic rich fractions (PRFs) from Indian high-altitude variety of *G. lucidum*. Aqueous extracts as well as diethyl ether, ethyl acetate, and residual phenolic fractions were prepared from *G. lucidum* mycelia and fruiting body. All these extracts and fractions were characterized by various antioxidant assays and high-performance thin layer chromatography (HPTLC). The extracts and fractions were also evaluated for potential bioefficacy, i.e., antibacterial activities against three major bacterial pathogens, namely, *Escherichia coli* (*E. coli*), *Salmonella typhi* (*S. typhi*), and *Staphylococcus aureus* (*S. aureus*), and protective effect against hypoxia stress on the human embryonic kidney HEK 293 cell lines.

## 2. Materials and Methods

### 2.1. Chemicals and Reagents

All chemicals and reagents used in the experimental procedures were purchased from Sigma-Aldrich (USA) and belonged to HPLC grade. Water used for maceration, preparation of extract, and in other purposes was of Millipore grade (Merck, USA).

### 2.2. Bacterial Strains and Cell Lines

The microbial strains used were *E. coli* (ATCC 9837), *S. typhi* (clinical isolate from All India Institute of Medical Sciences, Delhi, India), and *S. aureus* (ATCC 12600). Cell line used was the human embryonic kidney HEK 293 cell line.

### 2.3. Preparation of Aqueous Extracts of *G. lucidum* Mycelium and Fruiting Body

*G. lucidum* mycelium (GLM) and fruiting body (GLF) powder were commercially procured from M/s Aryan Enterprises, Delhi, India (batch numbers GLMP-1603 and GLFP-1603, respectively). Aqueous extracts of GLM and GLF were prepared by maceration. Briefly, in separate experimental settings, 500 grams each of GLM and GLF powder were dissolved in 5 L of water, accompanied by intermittent stirring. A small quantity of absolute ethanol was added to prevent contamination, and the vessel was covered and kept undisturbed at room temperature. After 2 days, the aqueous phase was filtered and centrifuged, and the supernatant was collected. The supernatant was concentrated by freeze-drying in a lyophilizer to obtain pure extracts (GLMaq and GLFAq) which were preserved at 4°C for further use.

### 2.4. Preparation of Phenolic Rich Fractions from Aqueous Extracts of GLM and GLF

Phenolic rich fractions were prepared from GLMaq and GLFaq by solvent fractionation technique using diethyl ether and ethyl acetate. In this method, GLMaq and GLFaq were dissolved in water to obtain a sample concentration of 200 g/L. Then, diethyl ether was added to the solution and kept on intermittent shaking. The fraction was separated using a separating funnel, following which two distinct layers, i.e., organic layer and aqueous layer, were formed. After complete fractionation, the upper organic layer was separated and the aqueous phase was introduced to a rotary evaporator to remove traces of diethyl ether. Furthermore, the process was repeated by mixing the aqueous layer with ethyl acetate, to obtain the second phase of fraction. Similarly as above, the resultant upper organic layer was separated, and traces of ethyl acetate were removed in a rotary evaporator to leave behind the residual phenolic fractions. All the three fractions obtained were then appropriately labeled (diethyl ether fractions: GLMdee and GLFdee; ethyl acetate fractions: GLMea and GLFea; and residual phenolic fractions: GLMph and GLFph), lyophilized, and preserved at 4°C for further use. All the aforementioned six phenolic rich fractions were collectively termed as “PRFs.”

### 2.5. Determination of Antioxidant Capacities

#### 2.5.1. Total Phenolic Content (TPC)

Total phenolic content (TPC) was determined according to a method reported by Kumar et al. [12]. In this assay, different concentrations of gallic acid, ranging from 1 mg/ml to 1 *µ*g/ml, were taken to plot the standard curve. TPC values of GLMaq, GLFaq, and PRFs were expressed as microgram gallic acid equivalent (GAE) per gram of extract (*µ*g GAE/g of extract). Absorbances were measured at 725 nm using a microtiter plate reader. All readings were taken in triplicates.

#### 2.5.2. Total Flavonoid Content (TFC)

Total flavonoid content (TFC) was determined according to a method described elsewhere [12]. Briefly, different concentrations of rutin ranging from 1 mg/ml to 1 *µ*g/ml rutin were taken as standards. TFC values of GLMaq, GLFaq, and PRFs were expressed as microgram rutin equivalent per gram of extract (*µ*g rutin/g of extract). Absorbances were measured at 510 nm, and analysis was performed in triplicates.

#### 2.5.3. Ferric Reducing Antioxidant Power (FRAP) Assay

FRAP of the various GLMaq, GLFaq and PRFs, was determined using a method described previously [13]. Here, different concentrations of trolox ranging from 400 *µ*M to 12.5 *µ*M were taken as standards. FRAP values of GLMaq, GLFaq, and PRFs were expressed as *µ*M trolox equivalent per g of extract (*µ*M trolox/g of extract). The absorbances were measured at 593 nm, and analysis was performed in triplicates.

#### 2.5.4. ABTS Assay

ABTS (2,2-azino-bis(3-ethylbenzothiazoline)-6-sulfonic acid) free-radical scavenging assay was carried out as described previously [13]. Different concentrations of trolox ranging from 800 *µ*M to 50 *µ*M were taken as standards. ABTS free-radical scavenging values of GLMaq, GLFaq, and PRFs were expressed as *µ*M trolox equivalent per gram of extract (*µ*M trolox/g of extract). Absorbances were recorded at 734 nm, and analysis was performed in triplicates.

### 2.6. Characterization by HPTLC

#### 2.6.1. HPTLC Analysis of Nucleobases

Separation and quantification of nucleobases, namely, thymine (T), uracil (U), adenine (Ad), cytosine (C), and guanosine (Gs), present in GLMaq, GLFaq, and PRFs was accomplished by HPTLC (Camag assembly, Switzerland) using a mobile phase described by Mishra et al. [14]. This mobile phase comprised dichloromethane, methanol, and formic acid in a ratio of 8 : 2.25 : 0.8. Separation was brought about on glass-backed silica gel 60 F_254_ HPTLC plates. The plate was developed at room temperature in a CAMAG twin-trough vertical development chamber, wherein the solvent front was maintained at 85 mm. Following development, the plate was subjected to densitometric scanning at a wavelength of 254 nm in the absorption mode, with deuterium as the light source. The quantities of the nucleobases as identified in the various extracts were calculated from the corresponding peak areas generated by winCats software (version 1.4.4.6337).

#### 2.6.2. HPTLC Analysis of Flavonoids

Separation of flavonoids, namely, quercetin (Q), gallic acid (G), ascorbic acid (As), and rutin (R), present in GLMaq, GLFaq, and PRFs was conducted using HPTLC (Camag assembly, Switzerland). The mobile phase used here was ethyl acetate : dichloromethane : formic acid : glacial acetic acid : methanol in a ratio of 10 : 10 : 1 : 1 : 2 [9]. Analytical parameters for separation and quantification of flavonoids were retained as described in Section 2.6.1.

### 2.7. Antibacterial Efficacy

#### 2.7.1. Preparation of Bacterial Inoculums

Glycerol stocks of three bacterial strains: *E. coli*, *S. typhi*, and *S. aureus* were revived by incubating them in nutrient broth for 16 to 18 h at 37°C, in an incubator-cum-shaker (Orbitek, India), at 90 rpm. Pure culture obtained was plated on a nutrient agar medium using the streak plate method, and the plates were preserved at 4°C for further experimentation.

#### 2.7.2. Screening of Fractions for Antibacterial Activity

The inhibitory activity of GLMaq, GLFaq, and PRFs against the aforementioned bacterial pathogens was determined by Kirby–Bauer disk diffusion methodology [15]. Here, 100 *µ*g of the GLMaq, GLFaq, and PRFs prepared in nutrient broth were loaded onto disks made out of Whatman No.1 filter paper and were appropriately dried under sterile conditions. These disks were placed upon nutrient agar medium plates swabbed with bacterial strains. The plates were incubated at 37°C for 16 to 18 h in an incubator-cum-shaker (Orbitek, India), at a shaking speed of 90 rpm, following which potential zones of inhibition around the disks were observed. Disks loaded with 5 *µ*g of kanamycin were taken as positive control for all the three bacterial pathogens under consideration.

#### 2.7.3. Determination of Minimal Inhibitory Concentration

16 to 18 h-old bacterial colonies cultured upon the nutrient agar medium were diluted in 0.8% physiological saline to prepare a 0.1 McFarland suspension. Then, the bacterial cultures were inoculated in test tubes containing 5 ml nutrient broth. Different concentrations ranging from 10 to 100 *µ*g of the specific GLF and GLM aqueous extracts and PRFs showing sufficient zones of inhibition were added to the nutrient broth. The tubes were then incubated at 37°C for 16 to 18 h in an incubator-cum-shaker (Orbitek, India), at a shaking speed of 90 rpm. The lowest or minimal concentration of a sample showing inhibition of bacterial growth was adjudged to be its minimal inhibitory concentration (MIC) for a given pathogen [15]. Kanamycin and pure nutrient broth medium were used as the positive and negative controls, respectively.

#### 2.7.4. Generation of Reactive Oxygen Species within Bacterial Cells

Levels of ROS generated within a bacterial pathogen's cellular environment upon exposure to specific GLF and GLM aqueous extracts and PRFs screened for potent antibacterial activity were measured using 2',7'-dichlorofluorescein diacetate (DCFDA) [16]. Briefly, the bacterial cell density was adjusted to 10^5^ CFU/ml using 0.8% physiological saline, and the cultures were inoculated in nutrient broth tubes. Various concentrations of the samples close to their respective MIC values and above were added to these nutrient broth tubes. The tubes were then incubated at 37°C for 3 h. Following this, the cultures were centrifuged at 4°C for 15°min at a speed of 500° ×g. The supernatant obtained was treated with 50 *µ*M DCFDA and incubated for 1 h at 37°C, in dark. A control group not treated with DCFDA was taken as control. The amount of ROS generated in the microbial samples was measured using a Cary Eclipse fluorescence spectrophotometer at an excitation wavelength of 485 nm and emission wavelength of 528 nm. All readings were taken in triplicates.

#### 2.7.5. Protein Leakage in Bacterial Cells

The protein leakage induced within the bacterial pathogen's cellular environment upon exposure to specific GLF and GLM aqueous extracts and PRFs screened for potent antibacterial activity was evaluated by Bradford's method using a protocol reported elsewhere [16, 17]. According to this method, 16 to 18 h cultures of the bacteria cultured in nutrient broth were washed with 0.8% physiological saline by centrifugation at 10,000 rpm for 20 min, followed by resuspension in saline. The bacterial cells were then treated with GLF and GLM fractions, with concentrations ranging from their respective MIC values and above. After incubation for 3 h, each bacterial suspension was centrifuged at 12,000 rpm for 15 min, and the supernatant obtained was analyzed for protein content using Bradford assay. Concentration of protein was estimated at a wavelength of 595 nm using bovine serum albumin (BSA) as standard. All readings were taken in triplicates.

### 2.8. Protective Effect against Hypoxia

The ameliorative effect of GLMaq, GLFaq, and PRFs on restoring cellular viability of human embryonic kidney (HEK) 293 cell lines under hypoxic stress conditions was evaluated. Herein, the HEK 293 cell lines were maintained in high glucose DMEM (Dulbecco's minimal essential medium) (pH 7.2), supplemented with antibiotics (gentamycin sulphate (100 mg/L) and penicillin (100 mg/L)) and enriched with foetal bovine serum (FBS) (10%, v/v) as described by Kirar et al. [18]. Cells were cultured in 96-well microtitre plates (Nunc, Denmark), maintaining a cell density of 10^5^ cells/cm^2^ and were kept in an incubator (Galaxy 170R, New Brunswick) with 21% O_2_, i.e., normoxic condition. Experiments were conducted on cells that were 70 to 80% confluent.

To assess the restorative action of the GLM and GLF extracts and PRFs, the experimental HEK 293 cells were divided into four groups for each type of the aforementioned sample. The grouping pattern was as follows: (i) normoxia control (N), (ii) normoxia supplemented with the GL extract or fraction under study (N + sample), (iii) hypoxia control (H), and (iv) hypoxia supplemented with the GL extract or fraction under study (H + sample).

Cells placed under normoxic conditions were maintained according to the parameters as described in preceding paragraphs, whereas, for subjecting to hypoxia stress, the HEK 293 cells were retained in a low oxygen environment (1% O_2_, 5% CO_2_, and 94.5% N_2_) for 24 h in a Galaxy 48R incubator (New Brunswick). For supplementation studies, different concentrations, i.e., 50, 75, 100, and 125 *µ*g/ml of the GLM and GLF samples were used. Cellular viability of the cells was evaluated using MTT assay [19]. The dose of a particular GL sample facilitating maximum restoration of cellular viability of the HEK 293 cell lines under hypoxic insult was inferred as its respective optimum dose.

### 2.9. Statistical Analysis

Statistical analysis for all the assays was carried out using SPSS software V.20 (IBM, USA). Statistical significance was verified at *P* < 0.05, for the data depicting dose-dependent antibacterial mechanisms of action.

## 3. Results and Discussion

### 3.1. Preparation of GL Aqueous Extracts and PRFs

Regarding aqueous extracts, GLMaq gave a higher percentage yield by weight (5.7%) than GLFaq (2.8%). This observation is in concurrence with previous reports where aqueous extract of *G. lucidum* mycelium, prepared by accelerated solvent extraction, gave better yield than *G. lucidum* fruiting body [13]. Between the diethyl ether fractions, GLFdee gave a fairly better yield (3.8%) than GLMdee (3.28%). In case of the ethyl acetate fractions, GLFea gave a much better yield (3.7%) than GLMea, which was only 0.64%. The residual phenolic fractions for both GLF and GLM gave exceedingly higher values, yielding 92.4% for GLFph and 92.16% for GLMph.

### 3.2. Antioxidant Capacity of GLM and GLF Aqueous Extracts and PRFs

#### 3.2.1. TPC Assay

**Figure 1:**
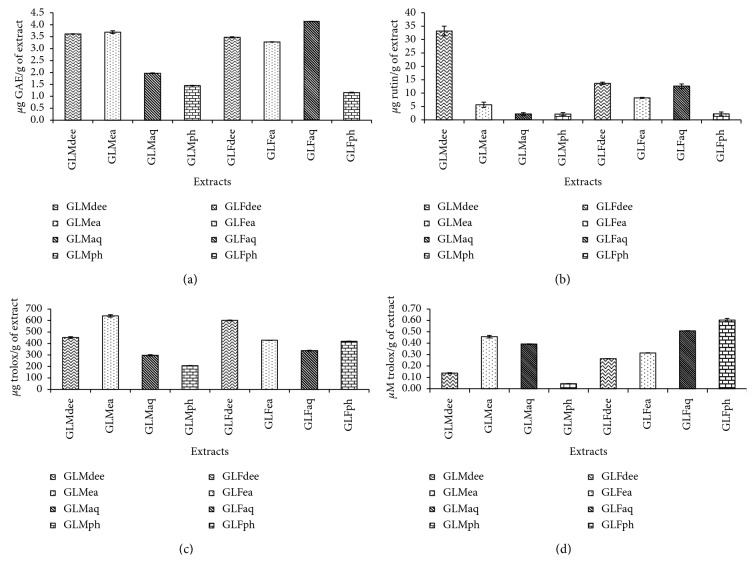
Total phenolic content (TPC) (a), total flavonoid content (TFC) (b), ferric reducing antioxidant power (FRAP) (c), and 2,2-azino-bis(3-ethylbenzothiazoline)-6-sulfonic acid (ABTS) free-radical scavenging potential (d) of aqueous extracts and PRFs of *Ganoderma lucidum* mycelium and fruiting body.

The total phenolic content of the GLMaq, GLFaq, and PRFs as estimated in terms of *μ*g GAE/g extract is shown (Figure 1(a)). GLFaq had the highest TPC content (4.13 *μ*g GAE/g of extract), followed by GLMea (3.69 *μ*g GAE/g of extract) and GLMdee (3.61 *μ*g GAE/g of extract).

#### 3.2.2. TFC Assay

TFC assay carried out for all the GLMaq, GLFaq, and PRFs brought out the highest total flavonoid content in GLMdee (54.89 *μ*g rutin/g of extract), followed by GLFdee (29.64 *μ*g rutin/g of extract) and GLFaq (27.86 *μ*g rutin/g of extract) (Figure 1(b)).

#### 3.2.3. FRAP Assay

FRAP assay proved GLMea to have the best antioxidant potency (640.5 *μ*M trolox/g of extract) in terms of ferric-reducing ability, closely followed by GLFdee (601 *μ*M trolox/g of extract) and GLMdee (452 *μ*M trolox/g of extract) (Figure 1(c)).

#### 3.2.4. ABTS Free-Radical Scavenging Assay

According to this assay, maximum ABTS free-radical scavenging activity was that of GLFph with a value of 0.6 *μ*M trolox/g of extract, followed closely by GLFaq (0.51 *μ*M trolox/g of extract) and GLMea (0.46 *μ*M trolox/g of extract) (Figure 1(d)).

Natural sources like fungi or plants are complex in nature and hence display variation in the types and quantities of bioactive compounds present in them, which may further be segregated into either primary or secondary class of metabolites [20]. In view of this complexity, it is preferable to evaluate the overall biological or therapeutic activity of a natural extract instead of individually studying the bioactive nature of each constituent metabolite. Thus, in the present study, representative antioxidant assays were carried out to establish the antioxidant potential of GLMaq, GLFaq, and PRFs. Though any particular trend pertaining to the antioxidant capacity of the phenolic fractions was not obtained, it was clearly observed that both the mycelium and fruiting body fractions possessed substantial antioxidant potential. Similar observations were acquired for aqueous, methanolic, and ethyl acetate fractions of *G. lucidum* where both mycelium as well as the fruiting body exhibited appreciable antioxidant activities [13].

Additionally, it can be stated here that the antioxidant potential of the diethyl ether and ethyl acetate fractions of GL mycelium and fruiting body in terms of TPC, TFC, and FRAP potential might be ascribed to the presence of numerous semipolar compounds in them. Such semipolar compounds have previously been reported to possess seemingly high antioxidant potential [13].

### 3.3. Characterization by HPTLC

#### 3.3.1. HPTLC Analysis of Nucleobases

**Figure 2:**
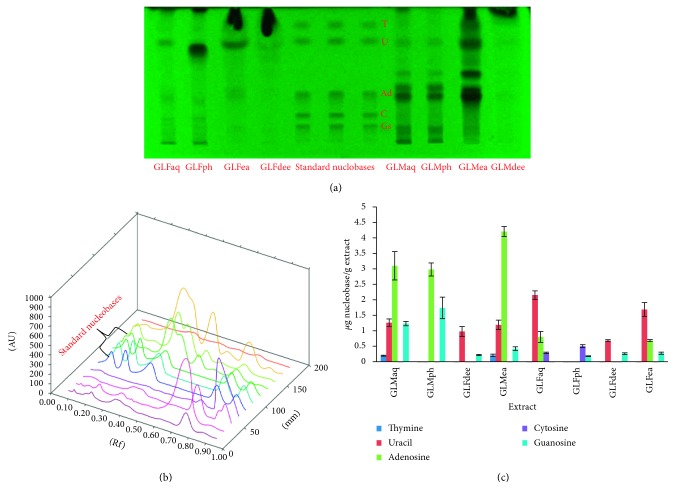
HPTLC chromatogram (a), 3D spectra (b), and quantities (c) of nucleobases identified in aqueous extracts and PRFs of *Ganoderma lucidum* mycelium and fruiting body (T: thymine; U: uracil; Ad: adenine; C: cytosine; Gs: guanosine).

Characterization of aqueous extracts and PRFs of GLF and GLM by HPTLC clearly proved the presence of two or more nucleobases in all the samples under study, as seen in the chromatogram (Figure 2(a)). GLMea and GLMaq fractions were the richest in terms of nucleobases containing thymine, uracil, adenine, and guanosine. GLFph was the least endowed fraction containing lower quantities of cytosine and guanosine. The peaks derived from corresponding 3D spectra are shown in Figure 2(b). The quantities of nucleobases present in GLMaq, GLFaq, and PRFS were calculated in terms of *μ*g nucleobase per gram of extract (Figure 2(c)). It is worthwhile to mention here that nucleobases like adenine, cytosine, and uracil have been reported to contribute significantly towards antifungal and antibacterial activities [21]. Thus, these results provided a lead to further investigate the antibacterial activity of the GL samples under study.

#### 3.3.2. HPTLC Analysis of Flavonoids

**Figure 3:**
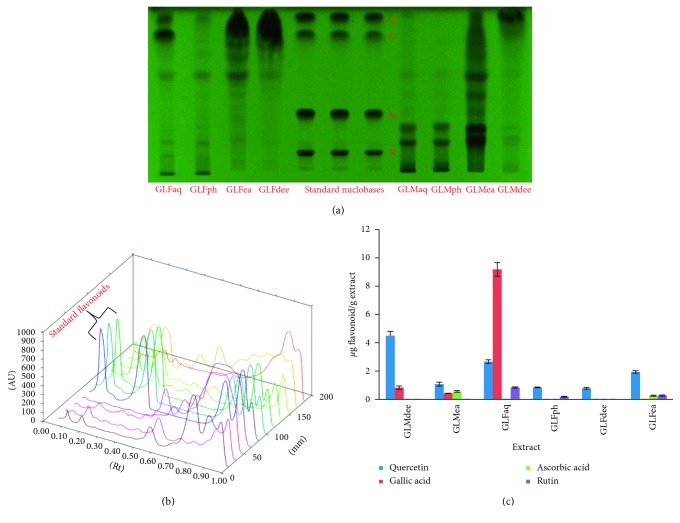
HPTLC chromatogram (a), 3D spectra (b), and quantities (c) of flavonoids identified in aqueous extracts and PRFs of *Ganoderma lucidum* mycelium and fruiting body (Q: quercetin; G: gallic acid; As: ascorbic acid; R: rutin).

HPTLC investigation of GLM and GLF aqueous extracts and PRFs confirmed the occurrence of one or more flavonoids in all the samples except GLMaq and GLMph as depicted in the HPTLC chromatogram (Figure 3(a)). It was observed that GLFaq was the most endowed extract, consisting of quercetin, gallic acid, and rutin. GLMdee had appreciable quantities of quercetin and gallic acid. GLMea contained lesser quantities of quercetin, gallic acid, and ascorbic acid. The peaks derived from corresponding 3D spectra are shown in Figure 3(b). The quantities of flavonoids present in GLMaq, GLFaq, and PRFS were calculated in terms of *μ*g flavonoid per gram of extract (Figure 3(c)).

Further, it can be stated that the appreciable flavonoid contents in GLMdee, GLMea, and GLFaq fractions can be correlated to the significant antioxidant potential of these fractions, as described in previous sections. This is in concurrence with the observations provided in earlier studies that higher quantities of flavonoids are responsible for better antioxidant behavior of natural extracts [22].

A previous study [21] had reported the inhibitory action of flavonoids like coumarin, quercetin, and kaempferol against bacterial pathogens, viz., *Staphylococcus aureus* and *Pseudomonas* spp. Owing to the confirmation of similar flavonoids in GLMaq, GLFaq, and PRFs under study, the antibacterial activity of these samples was investigated.

### 3.4. Antibacterial Efficacy

#### 3.4.1. Screening of GL Aqueous Extracts and PRFs for Antibacterial Activity and Determination of MIC

Aqueous extracts and PRFs of GLF and GLM demonstrated variable degrees of antibacterial activities on the pathogenic bacterial strains being studied. Overall, it was observed that *E*. *coli* was fairly inhibited by GLMaq, GLMph, GLMdee, GLMea, GLFph, and GLFdee. *S*. *typhi* was inhibited by almost all the fractions except GLFdee and GLFea. Antibacterial action was much less effective on *S. aureus* as compared to the aforementioned two pathogens.  Table 1 specifies the zones of inhibition in millimetre (mm) and corresponding MIC values in micrograms (*µ*g).

#### 3.4.2. ROS Generation by GL Extracts and PRFs in Pathogenic Bacteria

DCFDA is a fluorogenic dye used to measure hydroxyl, peroxyl, and ROS activity within cellular environment on account of its easy permeability within cells. This assay relies on the principle that, after diffusion into the cell, DCFDA gets deacetylated by cellular esterases to a nonfluorescent compound, which is later oxidized by ROS into 2',7'-dichlorofluorescein (DCF). DCF, being a highly fluorescent compound, can be detected by fluorescence spectroscopy with maximum excitation and emission spectra of 495 nm and 528 nm, respectively [17].

**Figure 4:**
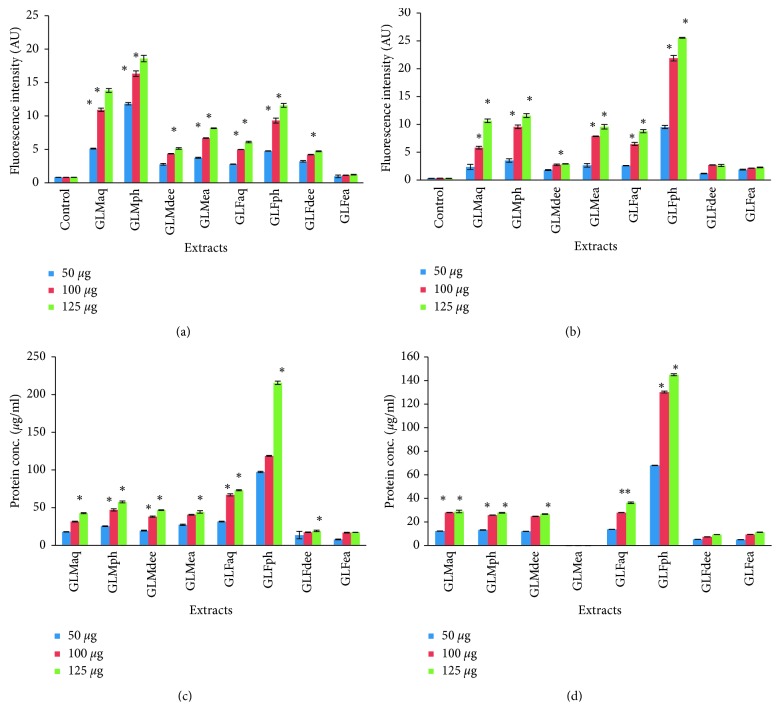
ROS generation in *Escherichia coli* (a), ROS generation in *Salmonella typhi* (b), protein leakage in *Escherichia coli* (c), and protein leakage in *Salmonella typhi* (d) induced by aqueous extracts and PRFs of *Ganoderma lucidum* mycelium and fruiting body (^*∗*^represents significant change at the level of *P* < 0.05).

Herbal extract-induced generation of free radicals, e.g., ROS, peroxide radicals, and superoxide radicals, within bacterial cells, leading to cellular membrane damage, has been reported to be a crucial antibacterial mechanism of action [17]. Keeping this in view, levels of ROS generated within the bacterial cells were estimated using DCFDA. As can be observed from Figure 4, almost all the GLM and GLF PRFs showed a positive linear trend for ROS generation in both *S. typhi* and *E. coli*, incrementing over a concentration range of 50 to 125 *μ*g/ml of the fractions. However, this trend was not observed in the case of GLFea, where concentration increase did not seem to be very effective in ROS generation. The best results were displayed by both the residual phenolic fractions, i.e., GLFph followed by GLMph.

#### 3.4.3. Protein Leakage Induced by GL Extracts and Fractions within Pathogenic Cells

The antibacterial mode of action of the GLM and GLF aqueous extracts and PRFs was further analyzed by investigating their effect on protein leakage within bacterial cells. Results as depicted in Figure 4 showed that, in the case of *E. coli,* there was a dose-dependent influence of extracts on induced protein leakage over a concentration range of 50 to 125 *μ*g/ml. The maximal protein leakage values were reported for GLFph and GLFaq. However, as per results obtained for *S*. *typhi*, the results were less effective than those for *E*. *coli*. GLMea did not have any apparent effect on protein leakage upon *S*. *typhi* cells. Here, GLFph and GLFaq showed the most substantial effect in inducing protein leakage within bacterial cells.

The better effectiveness of the GLM and GLF aqueous extracts and PRFs in inducing protein leakage within bacterial cells could be attributed to their rich content of flavonoids and nucleobases as verified from HPTLC analyses in the preceding sections. This observation can be corroborated with an earlier study stating that phytoconstituents exert appreciable influence on antimicrobial effect of natural extracts [23, 24].

### 3.5. Cytoprotective Effect against Hypoxia

It is already established in previous literature that hypoxic stress resulting due to renal diseases damages regulatory mechanisms and causes further progression of kidney disease. Low oxygen tension complicates the pathophysiology, sometimes leading to renal failure [25, 26].

Keeping this in perspective, the cytoprotective potential of GLMaq, GLFaq, and PRFs in imparting ameliorative action to HEK 293 cells, against hypoxia-induced cell death was evaluated in a dose-dependent manner where the concentration of the fractions ranged from 50 to 125 *µ*g/mL. It was observed that exposure to hypoxic stress induced cellular death by reducing cell viability to less than 40% in HEK 293 cell lines. This was in stark contrast to both the groups maintained under normoxia conditions (N and N + sample) where cell viability was ∼100%. However, upon supplementation with GLMph, GLMdee, GLFph, GLFdee, and GLFea, cell viability was observed to be substantially improved.

**Figure 5:**
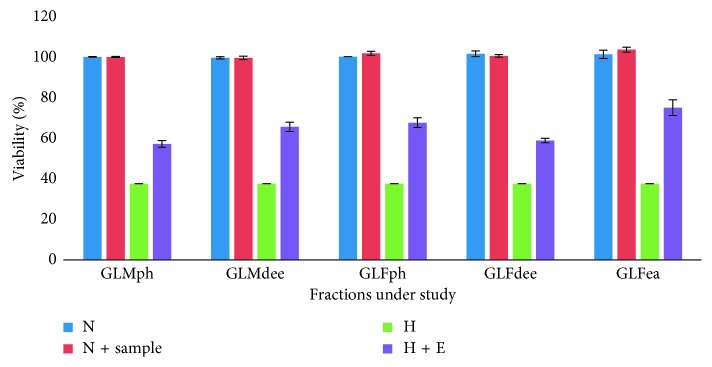
Cellular viability as determined by MTT assay. GLMph (100 *µ*g/ml), GLMdee (75 *µ*g/ml), GLFph (50 *µ*g/ml), GLFdee (100 *µ*g/ml), and GLFea (50 *µ*g/ml) improved cellular viability to 57%, 65.5%, 67.52%, 58.73%, and 75%, under hypoxia. N: normoxia, N + sample: normoxia supplemented with respective phenolic rich fraction of *Ganoderma lucidum* fruiting body or mycelium, H: hypoxia, H + E: hypoxia supplemented with respective phenolic rich fraction of *Ganoderma lucidum* fruiting body or mycelium (numbers in parentheses indicate the corresponding optimal dose for each phenolic rich fraction).

It was discerned that GLFea and GLFph fractions imparted maximum cytoprotective action by revamping HEK 293 cellular viability to 75% and 67.52%, respectively, at optimal doses of 50 *µ*g/mL. This was followed by GLMdee, GLFdee, and GLMph fractions which recuperated viability to 65.5%, 58.73%, and 57%, respectively. The optimal doses of GLMdee, GLFdee, and GLMph fractions were established to be 75, 100, and 100 *µ*g/mL, respectively (Figure 5). The ability of these fractions in modulating cell viability in stress could be accredited to the presence of nucleobases in them as described in preceding sections. This is in line with a previous study that reported the role of nucleobase supplementation in reduction of apoptosis [27].

## 4. Conclusion

The current study clearly indicates that the aqueous extracts and phenolic rich fractions prepared from *Ganoderma lucidum* fruiting body and mycelia displayed appreciable antioxidant and free-radical scavenging effects, as proven by various assays. Also, the fractions were proven to impart remarkable inhibitory potential against bacterial pathogens by instigating the generation of reactive oxygen species and protein leakage within the bacterial cells. This observation was in coherence with the presence of flavonoids and nucleobases in these fractions, which have been widely reported to possess antimicrobial efficacy. Some of the phenolic rich fractions markedly improved cellular viability of HEK 293 cells under hypoxic stress. These results collectively taken together could widen the prospect of using natural source-derived compounds as therapeutic solutions for the treatment of various diseases.

## Figures and Tables

**Table 1:** Diameters of inhibition zones (in mean (mm) ± SD) after treatment with aqueous extracts and PRFs of GLM and GLF; MIC values (in mean (*µ*g) ± SD), in parentheses.

	GLMaq	GLMph	GLMdee	GLMea	GLFaq	GLFph	GLFdee	GLFea
*S. typhi*	8 ± 0.12 (25 ± 1.75)	8 ± 0.55 (30 ± 2.1)	8.5 ± 0.47 (45 ± 3.15)	6.3 ± 0.15 (60 ± 7.12)	8.2 ± 0.76 (65 ± 3.46)	7 ± 0.32 (30 ± 1.08)	—	—
*E. coli*	7.5 ± 0.3 (35 ± 2.36)	8.6 ± 0.5 (30 ± 4.52)	6.5 ± 0.11 (55 ± 3.81)	6.5 ± 0.15 (55 ± 1.18)	—	7.4 ± 0.1 (40 ± 2.19)	6.5 ± 0.28 (55 ± 2)	—

## Data Availability

The data used to support the findings of this study are available from the corresponding author upon request.
